# Diverse ruthenium nitrides stabilized under pressure: a theoretical prediction

**DOI:** 10.1038/srep33506

**Published:** 2016-09-15

**Authors:** Yunkun Zhang, Lailei Wu, Biao Wan, Yangzheng Lin, Qingyang Hu, Yan Zhao, Rui Gao, Zhiping Li, Jingwu Zhang, Huiyang Gou

**Affiliations:** 1 Key Laboratory of Metastable Materials Science and Technology, College of Material Science and Engineering, Yanshan University, Qinhuangdao 066004, China; 2 Center for High Pressure Science and Technology Advanced Research, Beijing 100094, China; 3 Geophysical Laboratory, Carnegie Institution of Washington, 5251 Broad Branch Road NW, Washington, DC 20015, USA; 4 Key Laboratory of Applied Chemistry, College of Environmental and Chemical Engineering, Yanshan University, Qinhuangdao 066004, China

## Abstract

First-principles calculations were performed to understand the structural stability, synthesis routes, mechanical and electronic properties of diverse ruthenium nitrides. RuN with a new *I*-4*m*2 symmetry stabilized by pressure is found to be energetically preferred over the experimental NaCl-type and ZnS-type ones. The *Pnnm*-RuN_2_ is found to be stable above 1.1 GPa, in agreement with the experimental results. Specifically, new stoichiometries like RuN_3_ and RuN_4_ are proposed firstly to be thermodynamically stable, and the dynamical and mechanical stabilities of the newly predicted structures have been verified by checking their phonon spectra and elastic constants. A phase transition from *P*4/*mmm*-RuN_4_ to *C*2/*c*-RuN_4_ is also uncovered at 23.0 GPa. Drawn from bonding and band structure analysis, *P*4/*mmm*-RuN_4_ exhibits semi-metal-like behavior and becomes a semiconductor for the high-pressure *C*2/*c*-RuN_4_ phase. Meanwhile the *P*2_1_/*c*-RuN_3_ shows metallic feature. Highly directional covalent N-N and Ru-N bonds are formed and dominating in N-enriched Ru nitrides, making them promising hard materials.

The search of hard or superhard materials is of great interest due to the fundamental importance and technological applications^1^,^2^,^3^,^4^. Transition metal (TM) nitrides, because of the strong covalent bonding between TM and N atoms, are considered to be promising candidates for hard materials^5^,^6^,^7^,^8^. The inert nature of noble metals (*e.g.*, Os, Ir, Pt, Ru, Rh and Pd) used to hamper the reaction with nitrogen; however, the discovery of platinum pernitrides overcomes the chemical barrier^9^,^10^,^11^. Later on, iridium, osmium and palladium were also found to form pernitrides under pressure^12^,^13^,^14^,^15^. Recently, marcasite-type rhodium and ruthenium pernitrides have been successfully synthesized as well^16^,^17^. Among these nitrides, IrN_2_ was found to have bulk modulus of 428 GPa^12^,^18^, higher than most of previously synthesized materials. PtN_2_ and OsN_2_ also possess greater incompressibility with bulk modulus of 372 GPa^9^,^10^ and 358 GPa^12^,^18^, respectively, comparable to the traditional superhard materials (*e.g.*, diamond and *c*-BN). The synthesis of the noble metal nitrides is a milestone that has set the stage of paradigm of novel superhard materials.

Within the binary system of Ru and N, a cubic NaCl-type RuN with the lattice parameter of *a* = 4.445 Å was reported in the earlier study^19^. ZnS-type RuN thin films were also reported in the later studies^20^,^21^. Recently, RuN_2_ with marcasite-type structure and bulk modulus of 330 GPa was identified by Niwa *et al*.^17^. Despite of these efforts, the knowledge of basic structural type, mechanical and electronic properties of Ru nitrides is still lacking. Understanding this binary system requires comprehensive knowledge of their structural stability, composition changes, nitrogen bonding features and even pressure response. In this study, we are motivated to perform structural searching for stable phases in RuN_*x*_ (*x* = 1–4) in the pressure range of 0–50 GPa. We also examine the stability range of reported Ru nitrides and their phase transition under pressure. For Ru mononitrides, two structures (*I*-4*m*2-RuN and *R*-3*m*-RuN) are found to be more energetically stable than the previously reported NaCl-type and ZnS-type phases. With regard to Ru pernitrides, the stability of *Pnnm*-RuN_2_ is confirmed by phonon analysis and its behaviors under pressure are described. Moreover, Ru trinitrides and tetranitrides that have not yet been synthesized in laboratory were predicted with *P*2_1_/*c*-RuN_3_, *P*4/*mmm*-RuN_4_, *C*2/*c*-RuN_4_, and *Cmmm*-RuN_4_ structures, guiding further experimental attempts to produce N-rich Ru nitrides.

## Results and Discussion

**Figure 1:**
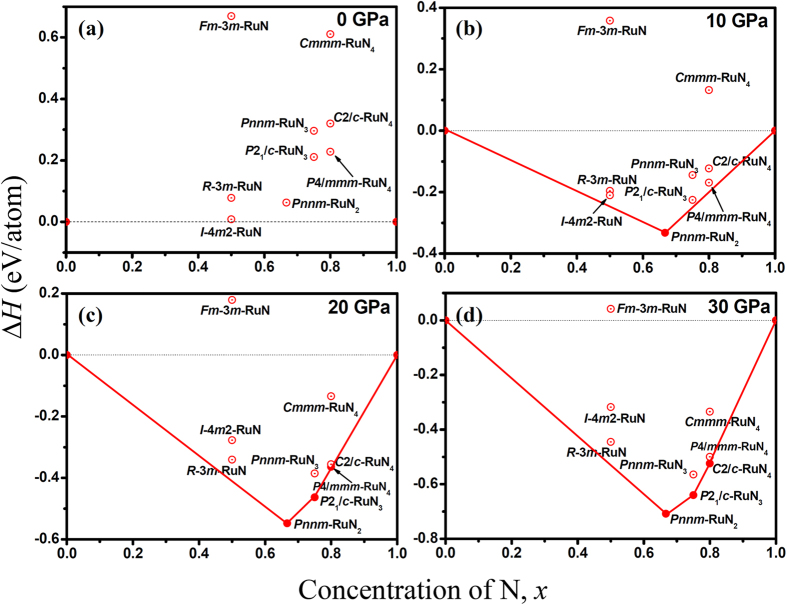
Formation enthalpies (∆*H*) of the structures of N-rich Ru-N binary compounds at pressures of (**a**) 0 GPa, (**b**) 10 GPa, (**c**) 20 GPa and (**d**) 30 GPa. The convex hulls connecting stable phases (solid circles) are shown by solid lines. Unstable/metastable phases are shown by open circles.

**Figure 2:**
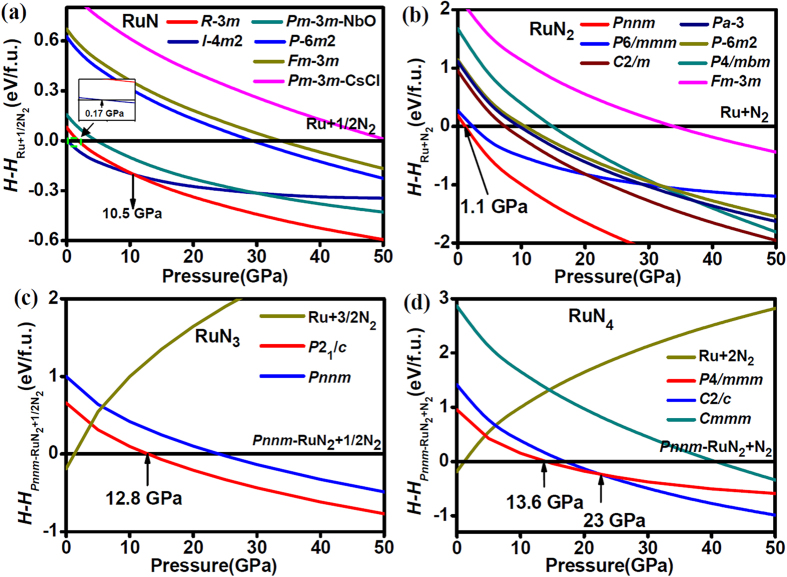
(**a,b**) Formation enthalpies of RuN and RuN_2_ with respect to Ru and nitrogen as a function of pressure, respectively. (**c,d**) Relative formation enthalpies of RuN_3_ and RuN_4_ with respect to *Pnnm*-RuN_2_ and nitrogen as a function of pressure, respectively.

**Figure 3:**
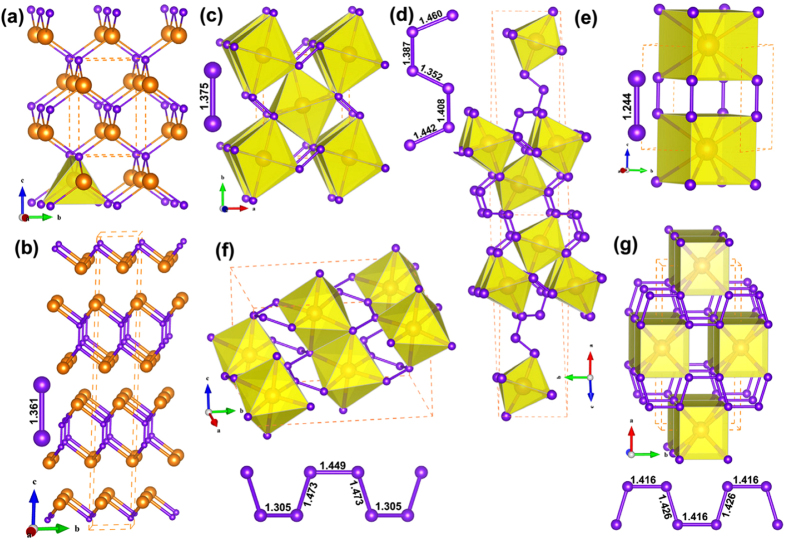
Crystal structures of Ru nitrides. (**a**) *I*-4*m*2-RuN with wyckoff position Ru (2*d*) (0, 0.5, 0.75) and N (2*a*) (0, 0, 0). (**b**) *R*-3*m*-RuN with wyckoff position Ru (6*c*) (0, 0, 0.2322) and N (6*c*) (0, 0, 0.0374). (**c**) *Pnnm*-RuN_2_ with wyckoff position Ru (2*a*) (0, 0, 0) and N (4*g*) (0.1236, 0.4058, 0). (**d**) *P*2_1_/*c*-RuN_3_ with wyckoff position Ru1 (4*e*) (0.5011, 0.1336, 0.1479), Ru2 (4*e*) (0.0313, 0.6289, 0.8824), N1 (4*e*) (0.6973, 0.3548, 0.1936), N2 (4*e*) (0.2439, 0.2475, 0.1333), N3 (4*e*) (0.3172, 0.9829, 0.1262), N4 (4*e*) (0.8148, 0.8595, 0.8150), N5 (4*e*) (0.2560, 0.7440, 0.8680) and N6 (4*e*) (0.1891, 0.4645, 0.8779). (**e**) *P*4/*mmm*-RuN_4_ with wyckoff position Ru (1*c*) (0.5, 0.5, 0) and N (4*i*) (0, 0.5, 0.3279). (**f**) *C*2/*c*-RuN_4_ with wyckoff position Ru (4*e*) (0, 0.0915, 0.25), N1 (8*f*) (0.2863, 0.0884, 0.6852) and N2 (8*f*) (0.3372, 0.2646, 0.2598). (**g**) C*mmm*-RuN_4_ with wyckoff position Ru (2*b*) (0, 0.5, 0) and N (8*q*) (0.1606, 0.1930, 0.5). The Ru and N atoms are represented as big orange and small purple spheres, respectively.

The NaCl-type RuN_*x*≈1_ (space group: *Fm*-3*m*) was suggested by Moreno-Armenta *et al*. using reactive pulsed laser ablation^19^, in which ruthenium target was laser ablated in N_2_ atmosphere in the pressure range of 1 × 10^−9^ to 0.16 Torr. Therefore, the NaCl-type RuN was initially chosen in our calculations. Interestingly, NaCl-type RuN is energetically unfavorable up to at least 50 GPa, as we can see from the convex hull of Ru-N system (Fig. 1) and relative formation enthalpies of RuN as a function of pressure (Fig. 2a). Furthermore, the calculated elastic constants (*C*_*ij*_) also rule out the NaCl-type RuN due to the mechanical instability with a negative *C*_44_ value (−94 GPa), according to the Born-Huang criterion^22^. Moreover, the phonon dispersion of NaCl-type RuN shows imaginary frequency in the Brillouin Zone (see Supplementary Fig. S1), indicating its dynamic instability. ZnS-type RuN was also reported to have been deposited by pulsed-DC magnetron sputtering at the pressure of 1 Pa and the temperature of about 50 °C^20^,^21^. Similar to NaCl-type RuN, we found that ZnS-type RuN is also mechanically unstable with a negative *C*_44_ value (−170 GPa). All the results strongly motivate us to search a possible ground state structure for RuN. Two candidates, the *I*-4*m*2-RuN and the *R*-3*m*-RuN, were survived in our structure searches. Interestingly, ZnS-type structure is relaxed to be an *I*-4*m*2-RuN symmetry, consistent with our structure searching results. Nevertheless, the formation enthalpy of *I*-4*m*2-RuN is positive below 0.17 GPa, as shown in Fig. 1. As the pressure increases, *I*-4*m*2-RuN becomes energetically favorable in the pressure range of 0.17 to 10.5 GPa, and then *R*-3*m*-RuN stands out up to 50 GPa (see Fig. 2a). Nevertheless, as shown in the convex hull, the predicted *I*-4*m*2-RuN has a very narrow stable range, and then both *I*-4*m*2-RuN and *R*-3*m*-RuN become metastable with the increasing pressure. In *I*-4*m*2-RuN, as demonstrated in Fig. 3a, N atoms occupy the center and vertex positions of the tetragonal lattices. Ru and neighboring N atoms constitute a tetrahedron with Ru atom situated in the center, and the tetrahedrons are connected by sharing vertex. The bond length of Ru-N is found to be 1.968 Å, but the separation of N-N is relatively large, 3.068 Å, limiting its capability of forming polynitrogen. For *R*-3*m*-RuN (see Fig. 3b), Ru and N atoms constitute a puckered 2D graphene-like honeycomb structure paralleling to the *xy* plane, in which the bond distance of Ru-N is 2.007 Å. The honeycomb structure was also observed in the IIB selenides and tellurides^23^. The puckered honeycomb sheets are connected by bridging N-N bonds forming a Ru-N-N-Ru layer, stacking along *c* axis. The N-N bond length in *R*-3*m*-RuN is 1.361 Å, shorter than the typical N-N single bond (1.45 Å in N_2_H_4_), but much longer than the double bond (1.21 Å for N_2_F_2_) and triple bond (1.09 Å for N_2_)^24^.

Recently, *Pnnm*-RuN_2_ was synthesized by direct chemical reaction between ruthenium and molecular nitrogen above the pressure of 32 GPa^17^. The ground-state structure of RuN_2_ was confirmed by the formation enthalpy curves as a function of pressure (Fig. 2b), and its formation enthalpy turns negative above 1.1 GPa. The convex hull also shows that *Pnnm*-RuN_2_ is the most stable phase in Ru-N phase diagram above the pressure of 10 GPa. In *Pnnm*-RuN_2_ (shown in Fig. 3c), Ru atoms are located in the center and vertex sites of the orthorhombic lattice. Each Ru atom is coordinated by six N atoms forming a RuN_6_ octahedron. The four equatorial and two axial Ru-N distances in RuN_6_ octahedron are 2.102 and 2.057 Å, respectively. The RuN_6_ octahedrons that situated in the center of the unit cell and the ones in the vertexes are connected by sharing corners and N_2_ dimers. The N_2_ dimer has a bond length of 1.375 Å, shorter than that in the OsN_2_ (~1.4 Å)^12^, IrN_2_ (1.42 Å)^18^ and PtN_2_ (1.41 Å)^10^, but larger than that in RhN_2_ (1.30 Å)^18^. The strong covalent N–N bonding in N_2_ dimer provides a strengthening effect on the elastic modulus.

It is known that IrP_3_^25^, IrAs_3_^26^, IrSb_3_^26^, CoP_3_^25^, and RhP_3_^25^ with cubic skutterudite CoAs_3_-type structure were synthesized in experiments. The chemical related compounds, including the corresponding nitrides IrN_3_^27^, CoN_3_^28^ and RhN_3_^28^ with the same type structure were also suggested by first-principles calculations. Besides, *Imm*2-TcN_3_, *P*4/*mmm*-TcN_4_, *Imm*2-ReN_3_ and *Cmmm*-ReN_4_ were also proposed by Zhao *et al*.^29^,^30^, together with ReN_4_, OsN_4_ and WN_4_ with ReP_4_-type structure by Aydin *et al*.^31^. To explore the possibility for Ru nitrides with higher nitrogen concentration, Ru trinitrides and tetranitrides were also searched in our calculations. Simultaneously, the calculated formation enthalpy-pressure curves with respect to *Pnnm*-RuN_2_ are given in Fig. 2c,d, respectively. For Ru trinitrides, a new *P*2_1_/*c* type structure for RuN_3_ is found to be favored over RuN_2_+1/2N_2_ above 12.8 GPa and thermodynamically stable up to at least 50 GPa (see Fig. 2c). *P*2_1_/*c*-RuN_3_ (Fig. 3d) contains two types of distorted RuN_6_ octahedrons and puckered S-shaped N_6_ units. In comparison with *Pnnm*-RuN_2_, *P*2_1_/*c*-RuN_3_ shows a variety of Ru-N distances from 1.976 to 2.275 Å for distorted RuN_6_ octahedrons. Besides, different with the regular RuN_6_ octahedron in *Pnnm*-RuN_2_, the distorted RuN_6_ octahedrons in *P*2_1_/*c*-RuN_3_ have the axis N-Ru-N angles of 166° and 170°, respectively. Moreover, in *Pnnm*-RuN_2_, there is only one unique N-N distance of 1.375 Å in N_2_ dimer, whereas the N-N distances in puckered N_6_ units in *P*2_1_/*c*-RuN_3_ vary from 1.352 to 1.460 Å. Interestingly, the stacking of RuN_6_ octahedrons in *P*2_1_/*c*-RuN_3_ becomes more packed than that in *Pnnm*-RuN_2_. Furthermore, N_2_ dimers (N_6_ units) in *Pnnm*-RuN_2_ (*P*2_1_/*c*-RuN_3_) is paralleling to *xy* (*yz*) planes, which will be reflected on the great incompressibility along *a* and *b* axis (*b* and *c* axis) as expected.

With further increasing N concentration, a *P*4/*mmm*-RuN_4_ for Ru tetranitrides becomes energetically preferable relative to RuN_2_+N_2_ at 13.6 GPa, and transforms to *C*2/*c*-RuN_4_ at 23 GPa (see Fig. 2d). For comparison, the relative enthalpy of *Cmmm*-RuN_4_ with respect to *C*2/*c*-RuN_4_ as a function of pressure is shown in Supplementary Fig. S2. The *C*2/*c*-RuN_4_ is thermodynamically favorable up to 101 GPa and transforms to *Cmmm*-RuN_4_, which is preferable at least up to 150 GPa. For *P*4/*mmm*-RuN_4_ (Fig. 3e), Ru atoms occupy the center of the top and bottom of the tetragonal unit cell and N_2_ dimers locate in the center of the four sides of the lattice. In this structure, each Ru atom is surrounded by eight N atoms, forming RuN_8_ cuboids. The intra-layer cuboids are connected by sharing edges, and the interlayer ones are connected by vertical N_2_ dimers. The Ru-N bond length in RuN_8_ cuboid is 2.161 Å and the N-N bond length in N_2_ dimer is 1.244 Å, close to the typical double bond (1.21 Å).

Similar to *P*2_1_/*c*-RuN_3_, *C*2/*c*-RuN_4_ (shown in Fig. 3f) is also composed of distorted RuN_6_ octahedrons and puckered S-shaped N_∞_ chains. The intra-layer RuN_6_ octahedrons are connected by sharing edges and the interlayer ones by the puckered N_∞_ chains that extend infinitely in the crystal. The Ru-N bond lengths in the distorted RuN_6_ octahedrons are between 1.993 to 2.203 Å, and the N-N bond lengths in the N_∞_ chains are 1.305, 1.449 and 1.473 Å, close to the 1.32, 1.39 and 1.43 Å in the spiral N_∞_ chains in *C*2/*c*-CsN_2_^32^. The transition from *P*4/*mmm*-RuN_4_ to *C*2/*c*-RuN_4_ accompanies a decrease of the coordination number of Ru atoms from 8 to 6. Resembling *P*4/*mmm*-RuN_4_, RuN_8_ cuboids are also formed in *Cmmm*-RuN_4_ (Fig. 3g), but different with the intra-layer edge-sharing cuboids and N_2_ dimers in *P*4/*mmm*-RuN_4_, the intra-layer RuN_8_ cuboids are face-sharing and the interlayer cuboids are connected by planar N_∞_ chains in *Cmmm*-RuN_4_. The Ru-N bond length in the cuboids is 2.197 Å and the N-N bond distances in the planar N_∞_ chains are 1.416 and 1.426 Å, close to the typical N-N single bond length (1.45 Å). Also, the phase transition sequence of these Ru nitrides can also be reflected by the total energy-volume curves, which are given in Supplementary Fig. S3.

The calculated equilibrium lattice parameters of Ru nitrides with different stoichiometries and their formation enthalpies at 0 GPa are listed in Table 1 in comparison with available data^17^,^18^,^33^. Our results agree well with the experimental lattice parameters within a maximum error of 1.0%, and also with the theoretical values, indicating the reliability of our calculations.

The mechanical stability of Ru nitrides is examined by calculating the individual elastic constants (see Table 2), all proposed phases are mechanically stable at 0 GPa with the satisfactions of Born-Huang stability criteria^27^. To evaluate the mechanical performance of Ru nitrides, the calculated bulk modulus (*B*), shear modulus (*G*), *B*/*G* ratio, Young’s modulus (*E*), Poisson’s ratios (*ν*), and Vicker’s hardness (*Hv*) are also listed in Table 2. The *Cmmm*-RuN_4_ has the highest *C*_11_ (738 GPa), comparable with that of IrN_2_ (739 GPa)^27^. *C*_22_ of *Pnnm*-RuN_2_ is 769 GPa, close to that of PtN_2_ (798 GPa)^34^. *P*4/*mmm*-RuN_4_ has a *C*_33_ value of 698 GPa, comparable to that of OsN_2_ (683 GPa)^35^. The elastic constant, *C*_44_, is another important parameter reflecting the hardness of material. Among these Ru-N compounds, *Cmmm*-RuN_4_ has the lowest *C*_44_ value (61 GPa), lower than that of RhN_2_ (80 GPa)^36^, but higher than that of PdN_2_ (43 GPa)^37^. *P*2_1_/*c*-RuN_3_ has the highest value of *C*_44_ (188 GPa), thereby a relatively strong shear strength.

Materials with high bulk modulus are expected to be strong in resisting uniform compression. As shown in Table 2, the calculated bulk modulus within GGA level is 298 GPa for *Pnnm*-RuN_2_, consistent with the previous theory 305.9 GPa^33^. This value is lower than the measured value 330 GPa^17^ and the calculated value 343 GPa^18^, caused by the difference between LDA and GGA methods^9^,^38^. The bulk modulus of *Pnnm*-RuN_2_ is higher than that of RhN_2_ (235 GPa)^16^, although lower than that of PtN_2_ (372 GPa)^9^,^10^, IrN_2_ (428 GPa)^12^, and OsN_2_ (358 GPa)^12^,^18^. Besides the highest bulk modulus, *Pnnm*-RuN_2_ also has the highest shear modulus (180 GPa), close to that of PtN_2_ (187 GPa)^34^, while *I*-4*m*2-RuN has the lowest *G* value (66 GPa). Except RuN and *Cmmm*-RuN_4_, the calculated hardness of the ground-state and high-pressure phases of Ru nitrides are between 20.1–23.4 GPa, belonging to the class of hard materials. Poisson’s ratio is an important parameter of directionality of the covalent bonding, and low Poisson’s ratio points to a high degree of covalency. For the *Pnnm*-RuN_2_, *P*2_1_/*c*-RuN_3_, *P*4/*mmm*-RuN_4_, *C*2/*c*-RuN_4_ and *Cmmm*-RuN_4_, *v* values are between 0.20 and 0.29, indicating their covalent bonding. The *B*/*G* ratio represents the ductility of the materials. The high (low) *B*/*G* ratio means that the material is ductile (brittle), and the critical value is about 1.75^39^. From Table 2 we can see that *I*-4*m*2-RuN, *R*-3*m*-RuN, and *Cmmm*-RuN_4_ are ductile and the others are brittle.

The dynamical stability of the newly predicted phases, *P*2_1_/*c*-RuN_3_, *P*4/*mmm*-RuN_4_, *C*2/*c*-RuN_4_, and *Cmmm*-RuN_4_, and metastable *I*-4*m*2-RuN and *R*-3*m*-RuN, together with the synthesized *Pnnm*-RuN_2_, is checked by calculating the phonon spectra (see Supplementary Fig. S4). All these phases are dynamically stable at 0 GPa with no imaginary frequency found throughout the Brillouin zone.

**Figure 4:**
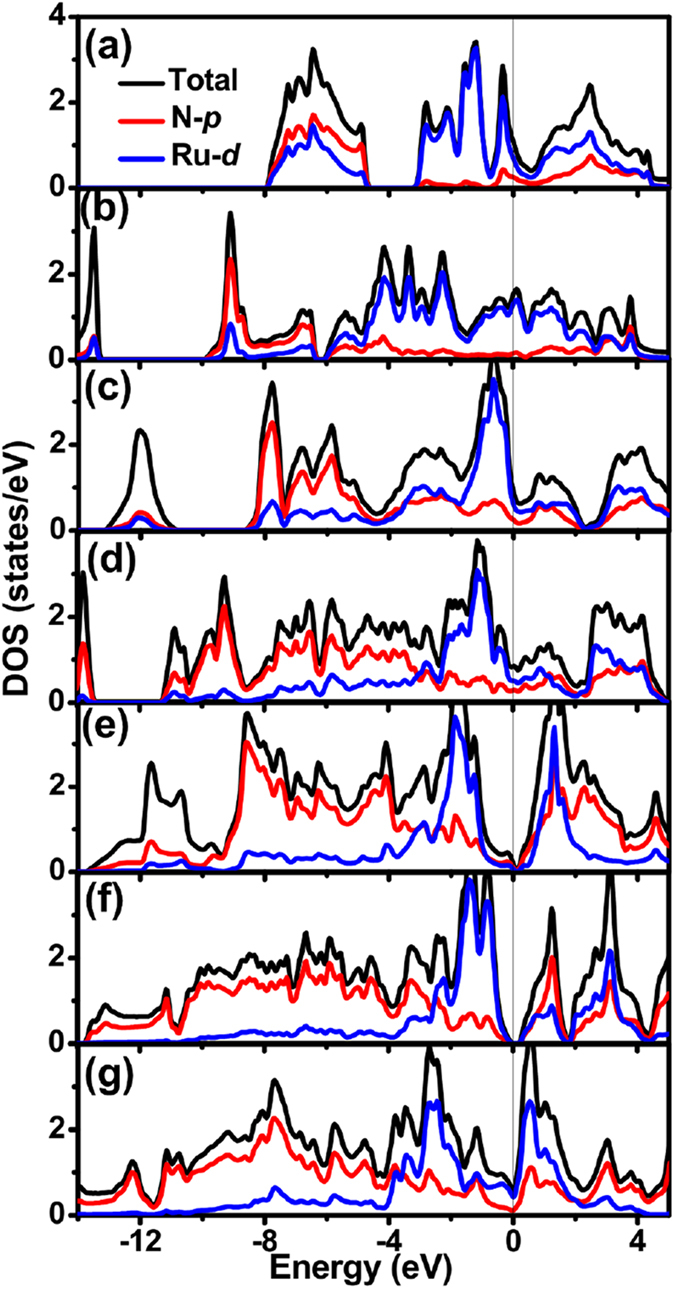
Calculated total and partial density of states for Ru nitrides. (**a**) *I*-4*m*2-RuN; (**b**) *R*-3*m*-RuN (**c**) *Pnnm*-RuN_2_; (**d**) *P*2_1_/*c*-RuN_3_; (**e**) *P*4/*mmm*-RuN_4_; (**f**) *C*2/*c*-RuN_4_ and (**g**) *Cmmm*-RuN_4_. The vertical dash line at zero is the Fermi energy level.

The total and partial density of states (DOS and PDOS) of Ru nitrides are plotted in Fig. 4 to understand the electronic properties and bonding features. It can be seen that except Ru tetranitrides, all of the compounds exhibit the metal features because of the finite DOS at the Fermi level (*E*_*F*_), which originates mostly from 4*d* electrons of Ru. Note that the significant hybridization between Ru 4*d* and N 2*p* orbitals is observed from about −8 to −5 eV in *I*-4*m*2-RuN, −10 to −5 eV in R-3*m*-RuN. For *Pnnm*-RuN_2_, the states located between about −8.5 eV and −4 eV mainly originate from N-2*p* orbitals with some contributions from Ru-4*d* states, and in the region from −4 eV to 2 eV, the Ru-4*d* states interacts mainly with the N-2*p* states. Furthermore, the arrangement of RuN_6_ octahedrons may derive Ru 4*d* orbitals splitting into triply degenerate *t*_*2g*_ orbitals at lower energy and doubly degenerate *e*_*g*_ orbitals at higher energy. Moreover, the pseudogap near the Fermi level is observed, enhancing the stability. For *P*2_1_/*c*-RuN_3_, in the range from −11.5 eV to −3 eV, the states are essentially dominated by N-2*p* orbitals due to the increase of N concentration, while from −3 eV to 2 eV, the states are mainly contributed by Ru-4*d* orbitals with admixture of N-2*p* orbitals.

**Figure 5:**
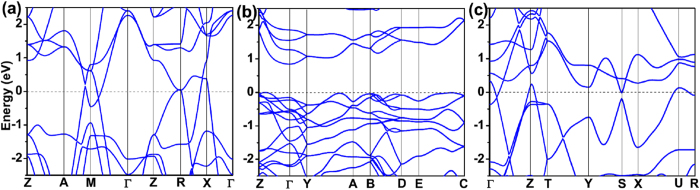
Band structure of (**a**) *P*4/*mmm*-RuN_4_; (**b**) *C*2/*c*-RuN_4_ and (**c**) *Cmmm*-RuN_4_. The *P*4/*mmm*-RuN_4_ and *Cmmm*-RuN_4_ have semimetallic feature, and *C*2/*c*-RuN_4_ is a semiconductor with an indirect band gap of 0.84 eV.

For Ru tetranitrides, Ru-4*d* orbitals and N-2*p* orbitals are hybridized in the region from about −3 eV to the Fermi level. The difference is that *P*4/*mmm*-RuN_4_ and *Cmmm*-RuN_4_ are semimetal-like while *C*2/*c*-RuN_4_ is semiconductor, which can be seen from their electronic band structure (Fig. 5). *P*4/*mmm*-RuN_4_ exhibits Dirac cones near the Fermi level in the A−M, M−Γ directions and at the R point of the Brillouin zone (BZ). *Cmmm*-RuN_4_ has a bulk Dirac cone below the Fermi level in the Γ−Z direction, giving rise to the small overlap of the valence band and the conduction band near the Γ point in the BZ. For *C*2/*c*-RuN_4_, the bottom of the conduction band is located at the Г point of BZ, and the top of the valence band at a point in the Z-Г direction, indicating semiconductor character with an indirect band gap of 0.84 eV. The band structures of the other Ru-N compounds are also computed and depicted in Supplementary Fig. S5, indicating their metallic character.

**Figure 6:**
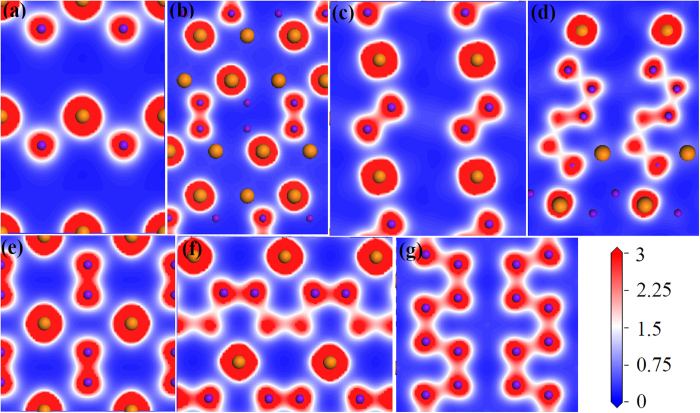
Calculated valence electron density distributions of Ru nitrides. (**a**) *I*-4*m*2-RuN in (100) plane; (**b**) *R*-3*m*-RuN in (110) plane; (**c**) *Pnnm*-RuN_2_ in (001) plane; (**d**) *P*2_1_/*c*-RuN_3_ in (101) plane; (**e**) *P*4/*mmm*-RuN_4_ in (010) plane; (**f**) *C*2/*c*-RuN_4_ in (001) plane and (**g**) *Cmmm*-RuN_4_ in (001) plane. The big orange and small purple spheres represent Ru and N atoms, respectively.

To gain deeper insights into the bonding nature in Ru-N compounds, we computed the distributions of valence electron density, as presented in Fig. 6. The Mulliken overlap populations (MOP) are also calculated to evaluate the relative bond strength. As we can see that for all these compounds, the nearly spherical charge distribution around Ru atoms indicates that the bonding between Ru and N atoms has partially ionic characteristic. As to *I*-4*m*2-RuN, the valence electrons are mainly located in the centre of Ru and N atoms, forming a zig-zag chain along *b* axis. The MOP for Ru-N bonding is 0.38, reflecting the moderate covalent bonding characteristic. The N-N distance is 3.068 Å, too far to form a covalent bonding. Different from *I*-4*m*2-RuN, σ covalent bonding of N-N are present in *R*-3*m*-RuN. The electron density at the N-N bond is higher with MOP of 0.83, indicating the strong N-N interactions along *c* axis. Compared with the N-N bonding, the Ru-N bonding is much weaker with a lower MOP value of 0.38, which is close to *I*-4*m*2-RuN.

For *Pnnm*-RuN_2_, the electron density is higher at the center of the N_2_ dimer with the MOP value of 0.76, resulting in the highest bulk modulus. The interactions between the Ru and N atoms are much weaker, as can be reflected by the MOP values 0.33 and 0.50 for Ru-N bonding. With regard to *P*2_1_/*c*-RuN_3_, the strong N-N covalent bonding can be found in the puckered N_6_ unit, with MOP values between 0.60 and 0.87. Due to the distortion and tilt of the RuN_6_ octahedrons, the length and strength of Ru-N bonding are irregular compared with *Pnnm*-RuN_2_ with MOP ranging from 0.28 to 0.47.

Similar to the *R*-3*m*-RuN and *Pnnm*-RuN_2_, N-N dimer in the *P*4/*mmm*-RuN_4_ is also characteristic of σ covalent bond, which contributes to a largest MOP value of 1.23, indicating the strong interactions between the N atoms. The largest *C*_33_ value comes from the contribution of strong directional covalent N-N bonding along *c* axis. While MOP for Ru-N bonding is 0.30, demonstrating weak interactions between Ru and N atoms. For *C*2/*c*-RuN_4_ and *Cmmm*-RuN_4_, the valence electrons are mainly located in the S-shaped N_∞_ chains. The difference is that Ru atoms are sandwiched in between two N_∞_ chains in *C*2/*c*-RuN_4_, while the Ru atoms and N_∞_ chains are not in the same plane in *Cmmm*-RuN_4_. The MOP is between 0.55 and 1.01 for N-N bonding, between 0.28 and 0.44 for Ru-N bonding in *C*2/*c*-RuN_4_, while the electronic density is in a narrow range for *Cmmm*-RuN_4_, with MOP values of 0.64 and 0.67 for N-N bonding, 0.27 for Ru-N bonding. Compared to *C*2/*c*-RuN_4_, the strength of the N-N bonding and the distribution of Ru-N bonding are more homogeneous in *Cmmm*-RuN_4_.

Nitrogen species in these compounds have various structural forms, such as single N atom, N_2_ dimers, N_6_ units and N_∞_ chains. Despite of the diverse features, the polynitrogens are in close correlation with the N-*sp* hybridization, characterized by σ covalent N-N bond. Mulliken charges analysis reveals a transferred charge of 0.55 *e* and 0.34 *e* from Ru to N atom for *I*-4*m*2-RuN and *R*-3*m*-RuN, respectively, 0.7 *e* from Ru to two N atoms for *Pnnm*-RuN_2_, 0.75 *e* from Ru to three N atoms for *P*2_1_/*c*-RuN_3_, 0.9 *e*, 0.81 *e*, and 0.83 *e* from Ru to four N atoms for *P*4/*mmm*-RuN_4_, *C*2/*c*-RuN_4_, and *Cmmm*-RuN_4_, respectively. The charge transfer also suggests the ionic feature of these compounds.

## Conclusions

In summary, we have systematically investigated the structures and properties of Ru nitrides at pressures of 0–50 GPa based on the density functional theory. We found two structures (*I*-4*m*2-RuN and *R*-3*m*-RuN) energetically prior to the previously reported NaCl-type and ZnS-type RuN. Besides the experimentally synthesized *Pnnm*-RuN_2_, new stoichiometries of *P*2_1_/*c*-RuN_3_, *P*4/*mmm*-RuN_4_, and *C*2/*c*-RuN_4_ are suggested for possible synthesis. The *P*2_1_/*c*-RuN_3_, *P*4/*mmm*-RuN_4_, and *C*2/*c*-RuN_4_ become stable at pressures above 12.8, 13.6, and 23 GPa, respectively. A new structure, *Cmmm*-RuN_4_ is also predicted to be stable above 101 GPa. The *Cmmm*-RuN_4_, *Pnnm*-RuN_2_, and *P*4/*mmm*-RuN_4_ possess the greatest incompressibility. The *Pnnm*-RuN_2_, *P*2_1_/*c*-RuN_3_, *P*4/*mmm*-RuN_4_, and *C*2/*c*-RuN_4_ are potential hard materials with the Vickers hardness between 20.1 and 23.4 GPa. The *P*4/*mmm*-RuN_4_ and *Cmmm*-RuN_4_ exhibit semi-metal-like properties and *C*2/*c*-RuN_4_ shows semiconductor features, while the *Pnnm*-RuN_2_ and *P*2_1_/*c*-RuN_3_ exhibit electronic characteristics of metals. Except *I*-4*m*2-RuN, high directional covalent N-N bonds are presented in all the other nitrides. A charge transfer from Ru to N atoms is predicted to occur, crucial to the stability of the Ru-N bonding in these compounds. These results are expected to stimulate the exploration and discovery of the newly predicted Ru nitrides, which may have practical technology applications due to their interesting mechanical and electronic properties.

## Methods

The stable structures of RuN_*x*_ (*x* = 1, 2, 3 and 4) with up to six (*x* = 1, 2) and four (*x* = 3, 4) formula units (f. u.) were searched at the pressures of 0, 20, 50 and 100 GPa using the particle swarm optimization method as implemented in the CALYPSO code^40^,^41^. Stable stoichiometries were determined by the construction of the convex hull: a compound is thermodynamically stable when its formation enthalpy falls on the line. The calculations of formation enthalpy and geometry optimizations presented in this study were carried out in the framework of density functional theory (DFT) with the Perdew-Burke-Ernzerhof (PBE) parameterization of the generalized gradient approximation (GGA)^42^ using CASTEP package^43^. An energy cutoff of 500 eV and dense *k*-point grids within the Monkhorst-Pack^44^ scheme were adopted for the sampling Brillouin zone of different structures, yielding excellent convergence for total energies (within 1 meV/atom). When the individual elastic constants were derived, the bulk (*B*), Young’s (*E*) and shear (*G*) moduli and Poisson’s ratio (*ν*) were obtained by using Voigt-Reuss-Hill approximation (VRH)^45^. The theoretical Vickers hardness was estimated by using the empirical model^46^, *H*_*v*_ = 2.0(*k*^2^*G*)^0.585^ − 3.0, where *k* = *G*/*B*. The global stability of Ru nitrides can be quantified by constructing the thermodynamic convex hull within considered pressures, which is defined as the average atomic formation enthalpy of the most stable phases at each composition:


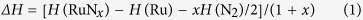


where *H* is the enthalpy of either a compound or a constituent element at a specific pressure. Here *α*-N_2_ and *ε*-N_2_ are adopted as the reference structure at below 10 GPa and 10–50 GPa for nitrogen, respectively. Phonon spectra of new proposed phases were calculated by finite displacement methods to examine their dynamical stabilities. The structures were visualized by VESTA^47^.

## Additional Information

**How to cite this article**: Zhang, Y. *et al*. Diverse ruthenium nitrides stabilized under pressure: a theoretical prediction. *Sci. Rep.* **6**, 33506; doi: 10.1038/srep33506 (2016).

## Supplementary Material

Supplementary Information

## Figures and Tables

**Table 1:** Calculated equilibrium lattice parameters, *a*, *b* and *c* (Å), *β* (deg.); formation enthalpies *ΔH* (eV/atom) of Ru nitrides at 0 GPa.

	S. G. (No.)	*a*	*b*	*c*	*β*	*ΔH*	Ref.
RuN	*I*-4*m*2(119)	3.068		4.929		0.008	
	*R*-3*m*(166)	2.835		18.220		0.078	
RuN_2_	*Pnnm*(58)	4.115	4.910	2.689		0.062	
		4.073	4.888	2.707			Exp^17^
		4.058	4.847	2.665			Cal^18^
		4.098	4.919	2.696			Cal^33^
RuN_3_	*P*2_1_/*c*(14)	11.925	4.070	13.032	154.12	0.211	
RuN_4_	*P*4/*mmm*(123)	3.6135		3.6137		0.263	
	*C*2/*c*(15)	3.834	8.888	5.720	117.83	0.355	
	*Cmmm*(65)	7.629	3.669	2.867		0.646	

**Table 2:** Calculated elastic constants *C*_*ij*_ (GPa), bulk modulus *B* (GPa), shear modulus *G* (GPa), *B*/*G* ratio, Poisson’s ratio *ν*, and Vickers hardness *Hv* (GPa) of Ru nitrides.

	S. G.	*C*_11_	*C*_22_	*C*_33_	*C*_44_	*C*_55_	*C*_66_	*C*_12_	*C*_13_	*C*_23_	*B*	*G*	*B/G*	*v*	*Hv*
RuN	*I*-4*m*2	371		305	99		37	177	234		260	66	3.94	0.38	—
	*R*-3*m*	406		530	87			205	173		271	105	2.58	0.33	—
RuN_2_	*Pnnm*	615	769	464	106	269	132	152	218	71	298	180	1.66	0.25	20.1
RuN_3_	*P*2_1_/*c*	367	473	639	188	186	201	183	127	127	253	174	1.45	0.22	23.4
RuN_4_	*P*4/*mmm*	283		698	104		129	112	38		174	128	1.36	0.20	20.9
	*C*2/*c*	540	664	344	151	208	286	199	172	97	257	171	1.50	0.23	22.1
	C*mmm*	738	496	374	61	73	257	132	184	135	269	133	2.02	0.29	12.3
