# Supplementary material for: Diverse ruthenium nitrides stabilized under pressure: a theoretical prediction
Source: Sci Rep. 2016 Sep 15;6:33506. doi: 10.1038/srep33506 (PMC5024155; doi:10.1038/srep33506)
Supplement: Supplementary Information [file srep33506-s1.pdf]

Supplementary information

Diverse ruthenium nitrides stabilized under pressure: a theoretical prediction

Yunkun Zhang,[1] Lailei Wu, [1]* Biao Wan,[1,2] Yangzheng Lin, [3] Qingyang Hu, [2, 3] Yan Zhao,[1] Rui Gao,[4] Zhiping Li,[4] Jingwu Zhang[1]* and Huiyang Gou[2]*

[1]Key Laboratory of Metastable Materials Science and Technology, College of Material Science and Engineering, Yanshan University, Qinhuangdao 066004, China.

[2]Center for High Pressure Science and Technology Advanced Research, Beijing 100094, China.

[3]Geophysical Laboratory, Carnegie Institution of Washington, 5251 Broad Branch Road NW, Washington, DC 20015, USA.

[4]Key Laboratory of Applied Chemistry, College of Environmental and Chemical Engineering, Yanshan University, Qinhuangdao 066004, China.

Correspondence and requests for materials should be addressed to

H. G. (huiyang.gou@gmail.com), L. W. (wll@ysu.edu.cn) and J. Z. (zjw@ysu.edu.cn)

Supplementary Figures

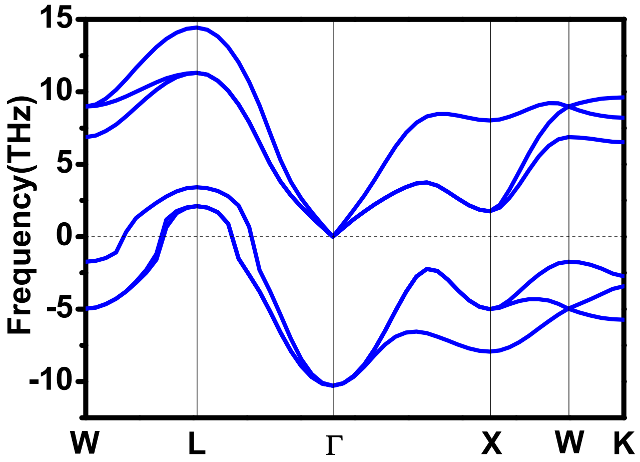

Figure S1. Phonon dispersion of NaCl-type RuN. Imaginary frequency in the Brillouin Zone indicates the dynamically instability of the compound. A supercell with 6×6×6 k-points were adopted.

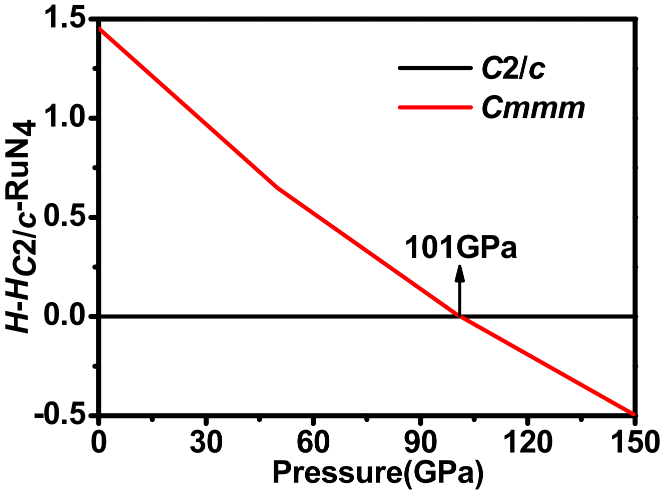

Figure S2. Relative enthalpies of *Cmmm*-RuN_4 with respect to *C*2/*c*-RuN_4 as a function of pressure.

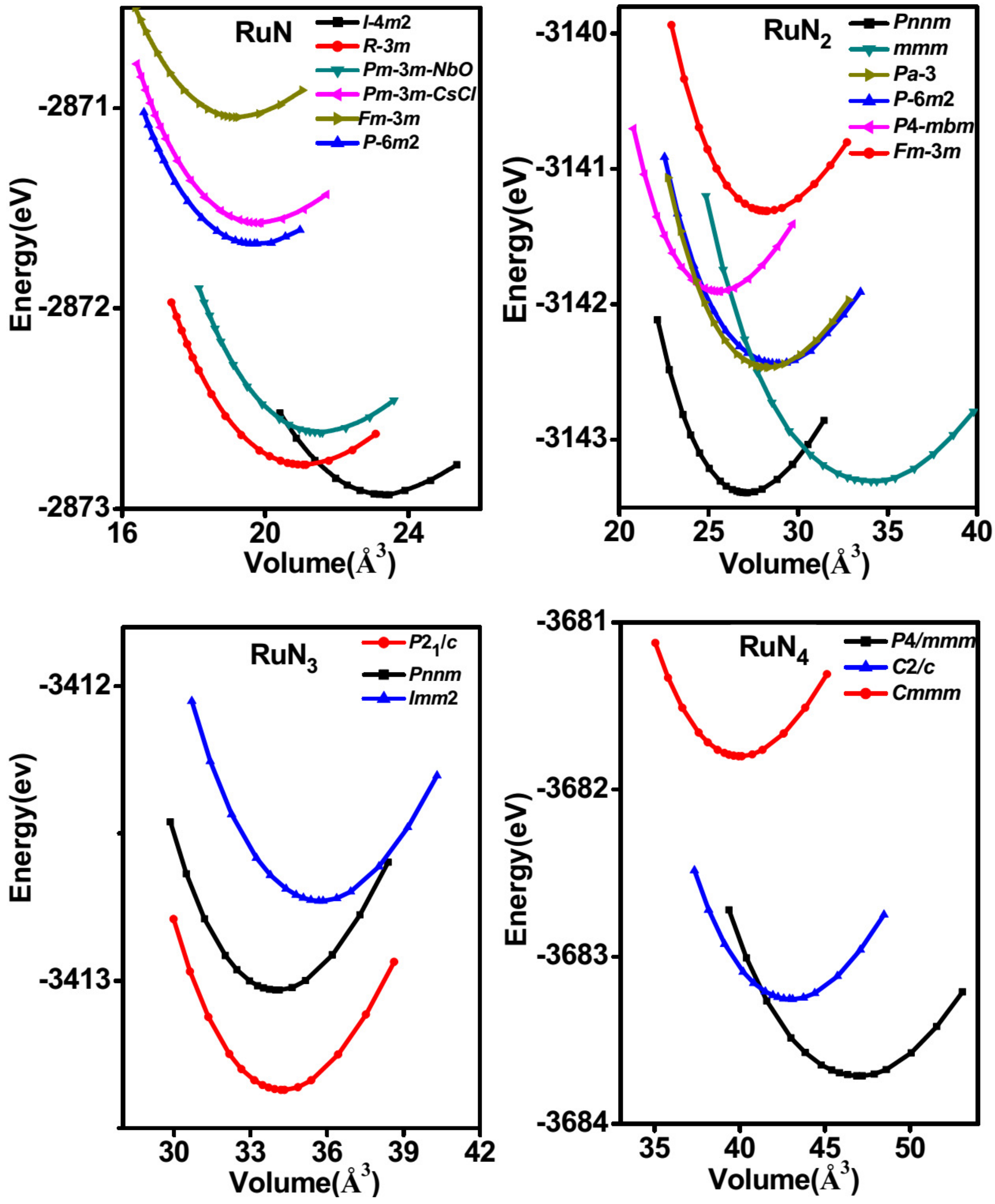

Figure S3. Total energy-volume curves of Ru nitrides.

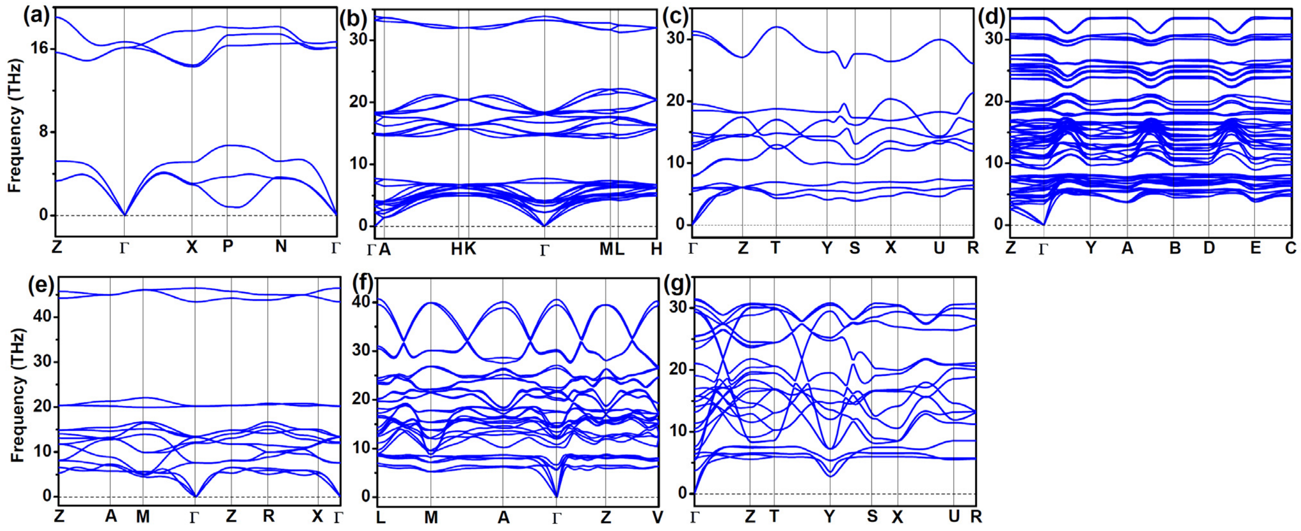

Figure S4. Phonon dispersion curves of Ru nitrides. (a) *I*-4*m*2-RuN; (b) *R*-3*m*-RuN (c) *Pnnm*-RuN_2; (d) *P*2$_1$/*c*-RuN_3; (e) *P*4/*mmm*-RuN_4; (f) *C*2/*c*-RuN_4 and (g) *Cmmm*-RuN_4. A supercell with 11×11×7 k-points for *I*-4*m*2-RuN, with 6×6×2 k-points for *R*-3*m*-RuN, with 3×3×5 k-points for *Pnnm*-RuN_2, with 3×3×2 k-points for *P*2$_1$/*c*-RuN_3, with 4×4×4 k-points for *P*4/*mmm*-RuN_4, with 4×4×3 k-points for *C*2/*c* -RuN_4, and with 2×4×5 k-points for *Cmmm*-RuN_4 were adopted.

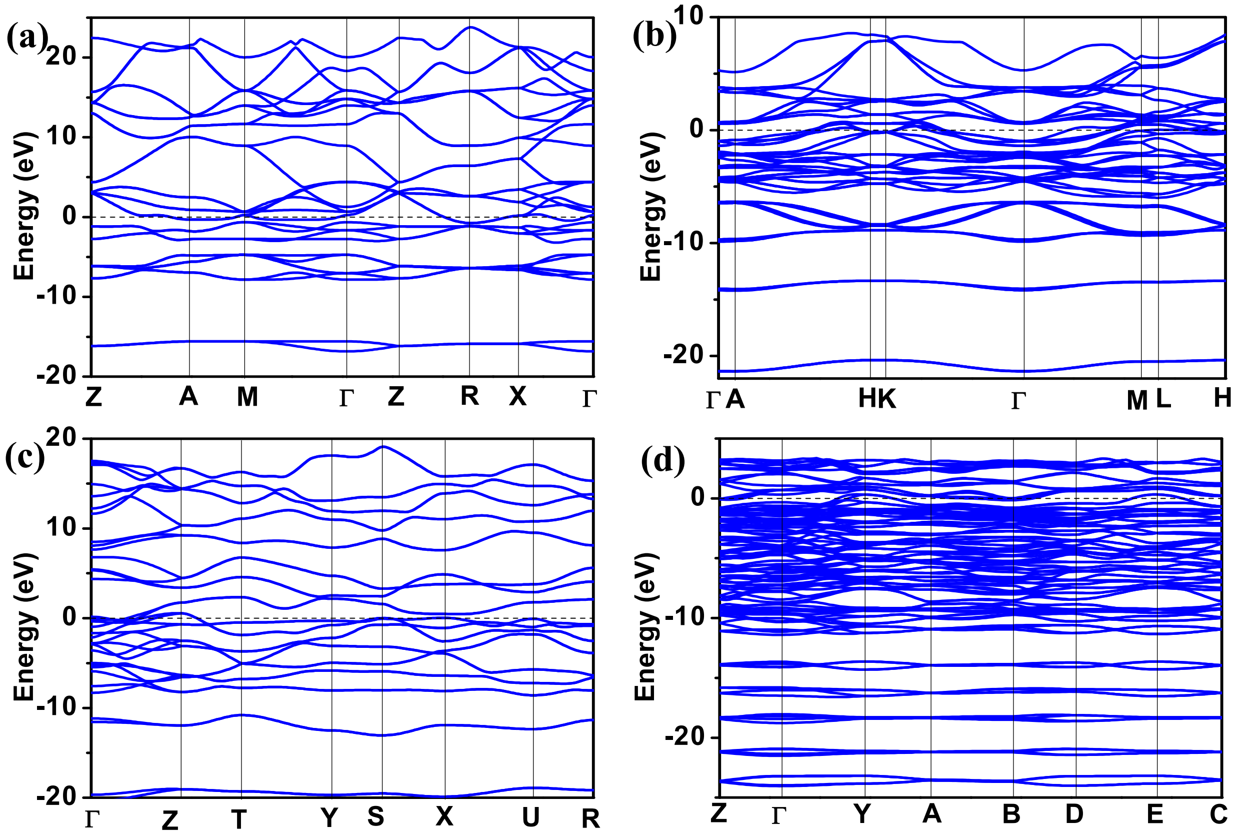

Figure S5. Band structure of (a) *I*-4*m*2-RuN; (b) *R*-3*m*-RuN; (c) *Pnnm*-RuN_2 and (d) *P*2$_1$/*c*-RuN_3.
